# Caries Activity and Its Associated Factors Among Primary Caregivers of Preschool Children

**DOI:** 10.1155/ijod/2925089

**Published:** 2026-09-12

**Authors:** Colin Tsz Wo Lee, Sifan Wang, Xinyi Zeng, Cynthia Kar Yung Yiu, Hai Ming Wong, Ni Zhou

**Affiliations:** ^1^ The University of Hong Kong, Hong Kong SAR, China, hku.hk; ^2^ Anning Maternal and Child Health Hospital, Anning, China

**Keywords:** caregiver, caries activity, children, dental caries, family, oral health

## Abstract

**Objectives:**

This study aimed to investigate the caries activity and associated factors among primary caregivers of preschoolers.

**Methods:**

This cross‐sectional study included 251 primary caregivers who were responsible for the daily care of preschool children. Caries activity of caregivers and children was evaluated using the Cariostat kit. Additionally, caregivers’ dental caries, oral hygiene status, oral health‐related behaviors, and sociodemographics were collected. Univariate and multivariate logistic regression were performed to identify independent factors.

**Results:**

The mean Cariostat score for participants was 1.57 ± 0.43, and 33.1% (*n* = 83) of caregivers had Cariostat score over 1.5. Primary caregivers who cared for children with high Cariostat scores were more likely to have high Cariostat scores themselves (*p* < 0.001). After adjustment, high caries activity was independently associated with DMFT ≥5 (OR = 2.31, 95% CI: 1.08–4.94), snacking ≥3 times/day (OR = 2.96, 1.41–6.28), grandparent caregivers (OR = 9.65, 2.18–68.70), and children under their care with high Cariostat scores (OR = 4.05, 2.09–7.97).

**Conclusion:**

Caregivers’ caries activity was associated with their own caries status, snacking frequency, and their children’s caries activity levels. Family‐based interventions targeting snacking consumption and caries management are recommended.

## 1. Introduction

Dental caries, a dynamic and preventable disease, is marked by the balance of remineralization and demineralization. Caries activity describes the incidence of dental caries and the rate of progression of existing lesions over a specified period [1]. While carious lesions are often detected visually or by tactile examination during clinical examination, it is also paramount for clinicians to anticipate upcoming caries activity; therefore, a comprehensive caries risk assessment is often needed to formulate individualized treatment plans for children [2]. Caries risk assessment involves a series of investigations, including individuals’ social and cultural background and their oral health‐related behaviors [3].

Dental caries is related to multiple factors. Evidence suggests that the initiation and progression of dental caries are linked to cariogenic pathogens such as *Streptococcus mutans* and *Lactobacilli* [4]. Other bacteria, for instance, *Leptotrichia*, *Selenomonas*, *Prevotella*, *Veillonella*, and *Neisseria*, were also associated with caries [5, 6]. Li et al. [7] suggested that caries occurrence was associated with children’s toothbrushing habits, the daily intake of cariogenic foods or drinks before bedtime, and caregivers’ oral health knowledge. Zou et al. [8] found that, within Chinese families, similar *S. mutans* genotypes were present in mothers and children, suggesting an association between children’s and their caregivers’ caries status.

The existing caries activity tests (CATs) can be classified into two major types: (i) estimation of cariogenic bacteria such as *Lactobacilli* and *S. mutans* in saliva or plaque and (ii) evaluation of virulent characteristics of microorganisms, such as acidogenesis or their ability to reduce chemical agents [9]. The Cariostat, a colorimetric test developed by Shimono and Sobue in 1974, uses a medium containing sucrose and two pH indicators to detect the continuous pH changes caused by bacteria in the plaque sample [10, 11]. Acid production from microbial sucrose metabolism lowers the medium pH, inducing a color change that reflects the acidogenicity of the plaque biofilm. Strong correlations between pH and Cariostat scores, and the high sensitivity of Cariostat in detecting caries, have been well documented [4, 11]. A longitudinal study revealed that Cariostat could predict the development of new carious lesions in preschool children [11]. However, while Cariostat measures plaque acidogenicity and cariogenic potential, it does not capture the full complexity of caries risk.

Other CAT kits have also been introduced in prior studies. Cariview, first developed in Korea, is a colorimetric caries activity test based on acid production by plaque bacteria [12]. Like Cariostat, Cariview is a plaque acidogenicity‐based test that indirectly estimates cariogenic potential. Additionally, Dentocult SM and Dentocult LB are CATs that can detect the density of *Streptococcus mutans* and *Lactobacilli*, respectively [13, 14]. A 2‐year longitudinal study found that Dentocult SM was more reliable than Dentocult LB for predicting caries progression [15]. A cross‐sectional study demonstrated that Cariview detected the acidogenic potential of the entire microorganism within dental plaques, exhibiting stronger correlations with preschool children’s *d*ft and *d*t indices than Dentocult SM did [12]. However, as a relatively new CAT, the number of studies examining its application remains limited, which restricts the evidence available for comparison. Conversely, Cariostat is a traditional and widely used CAT, with a substantial body of evidence supporting its validity and facilitating comparisons with previous studies [4, 11].

To date, although caries risk assessment and its related factors in young children have been extensively studied in preschool children, the caries risk of their primary caregivers remains underexplored. A recent case–control study revealed that caregivers of children with early childhood caries (ECC) had higher odds of dental caries than their peers, and children whose caregivers had caries tended to be affected by ECC [16]. As primary caregivers play a key role in maintaining oral health among young children, investigating their caries risk and related factors may inform the development of family‐based caries prevention strategies. Therefore, this study aimed to investigate the Cariostat score and its associated factors among caregivers of preschool children. Although the aforementioned CAT kits are noninvasive, chairside assessment methods that are relatively easy to perform, they do not directly detect existing carious lesions; instead, they evaluate the acidogenicity or cariogenic potential of the dental plaque. Those kits do not capture other risk determinants, such as dietary practices, fluoride exposure, or socioeconomic conditions. We hypothesize that caregivers’ Cariostat scores might be associated with their caries status, oral health‐related behaviors, and their children’s Cariostat scores.

## 2. Materials and Methods

### 2.1. Study Design and Participant Recruitment

This cross‐sectional study was conducted in Anning. Anning Maternity and Child Care Hospital provides regular annual dental check‐ups for local children, either at the hospital or at the kindergartens. Therefore, between March and November 2025, invitation letters were distributed to the primary caregivers of children who had dental examinations at the Anning Maternity and Child Care Hospital. Approximately half of the participants were recruited at the hospital, and the others were from the local kindergartens. This study was reviewed and approved by the local Ethics Committee of the Anning Maternity and Child Care Hospital (Ref No. ANFY2024KQ001) and the Institutional Review Board of the University of Hong Kong/Hospital Authority Hong Kong West Cluster (Ref No. UW24‐822). It was reported in accordance with the Strengthening the Reporting of Observational Studies in Epidemiology (STROBE) guidelines [17]. Caregivers who were willing to participate and provided written informed consent were enrolled in the study. The inclusion criteria included (i) the primary caregivers who were taking care of preschool children, living with the target child for at least 6 months; (ii) the cared children were 2–5 years of age; and (iii) participants without disabilities or other severe systemic diseases. The exclusion criteria were (i) participants who were unable to communicate normally or complete questionnaires and oral examinations; (ii) participants who were unwilling to join this study; and (iii) individuals who did not provide informed consent.

### 2.2. Sample Size Calculation

The primary outcome of this study was the Cariostat score of the primary caregivers who were responsible for the daily care of the preschoolers. In our pilot study, the average Cariostat score of parents was 1.38 ± 0.40. With a confidence level of 95%, *Z*_*α*/2_ was estimated to be 1.96, the standard deviation was 0.40, and the desired margin of error (*E*) was assumed to be ±0.05. Thus, at least 246 participants were required. Assuming a 90% response rate, 273 participants should be invited initially.

### 2.3. Data Collection

Data were collected through questionnaire surveys and oral examinations. After obtaining written informed consent, participants were asked to complete a structured, self‐administered questionnaire to collect information on sociodemographic characteristics (e.g., sex, age, educational level) and oral health behaviors (e.g., toothbrushing frequency, toothbrushing duration, snacking frequency). Toothbrushing frequency and duration were assessed using two questions: (1) “How many times do you brush your teeth per day?” (2) “How many minutes do you spend brushing your teeth each time?” Snack consumption frequency was assessed using one question: “How many times do you eat snacks between main meals per day?”

### 2.4. Dental Caries Assessment

Two calibrated, registered pediatric dentists conducted the dental examinations, with kappa values above 0.80. Dental caries status was evaluated in line with the coding criteria proposed by the World Health Organization [18]. The number of decayed, missing due to caries, and filled teeth (DMFT) scores was calculated, and the level of caries experience among the recruited caregivers was recoded into four levels based on their DMFT score, namely (i) very low caries experience with a DMFT score less than 5.0, (ii) low caries experience with a DMFT score of 5.0–8.9, (iii) moderate caries experience with a DMFT score of 9.0–13.9, and (iv) high caries experience with a DMFT score above 13.9 [18].

### 2.5. Oral Hygiene Assessment

Oral hygiene status was evaluated using the Simplified Debris Index (DI‐S) [19], with higher scores indicating poorer oral hygiene. Additionally, based on the DI‐S scores, oral hygiene status was classified into three levels, namely (i) good oral hygiene with a DI‐S score of 0–0.6; (ii) fair oral hygiene with a DI‐S score of 0.7–1.8; and (iii) poor oral hygiene with a DI‐S score of 1.9–3.0 [20].

### 2.6. Cariostat Test

The Cariostat kit (Gang Da Medical Technology Co., Ltd., China) was used to assess participants’ caries activity [21]. Several studies reported that Cariostat scores were significantly associated with caries status and salivary microbial communities [21–23]. A sterile swab was used to collect the plaque from the buccal surface of upper molars and lower anterior teeth in line with the manufacturer’s instructions. The collected samples were stored in a vial containing culture medium and incubated at 37 °C for 48 h. Two dental assistants (inter‐ and intra‐rater reliability above 0.80) compared the color of the medium to the reference chart provided in the Cariostat kit and recorded the scores, which ranged from 0 to 3 in 0.5‐point intervals. Cariostat scores were categorized into three levels: (i) low risk with a Cariostat score of 0–0.5; (ii) moderate risk with a Cariostat score of 1–1.5; and (iii) high risk with a Cariostat score of 2.0 or above [24].

### 2.7. Data Analysis

Statistical analyses were conducted using IBM SPSS Statistics 28.0 (IBM Corp, Armonk, New York). Ordinal data were analyzed using chi‐square tests and Fisher’s exact tests. Covariates with *p*‐values less than 0.1 were included in the univariate logistic regression model. Univariate and multivariate logistic regression models were used to examine crude and adjusted associations between potential risk factors and caries activity. Odds ratios (OR) and corresponding 95% confidence intervals (CI) were used to assess the associations. All the comparisons were two‐sided, and the significance level was set at 0.05.

## 3. Results

### 3.1. Characteristics of Participants

Among the invited primary caregivers, 251 received Cariostat assessment and completed the questionnaire survey (response rate: 93.7%). The caregivers were aged between 22 and 70 years old. Most primary caregivers were female (69.7%), with mothers comprising the predominant group at 66.1% and fathers at 28.3%. Regarding education, more than half (62.2%) of the primary caregivers held a bachelor’s degree or higher (Table 1).

**Table 1:** Characteristics of the recruited caregivers (*n* = 251).

Variables	*n* (%)
Gender	
Male	76 (30.3)
Female	175 (69.7)
Role in the family	
Mother	166 (66.1)
Father	71 (28.3)
Grandparents	14 (5.6)
Cariostat scores of children under care	
Low or moderate (0–1.5)	192 (76.5)
High (≥2.0)	59 (23.5)
Highest educational level in the family	
High school or below	95 (37.8)
Bachelor’s degree or above	156 (62.2)

### 3.2. Oral Health Status of the Recruited Caregivers

The oral health status of the 251 recruited primary caregivers was presented in Table 2. Regarding caries status, the mean DMFT index was 2.62 ± 3.31. The overall caries prevalence was 71.7%. When classified by caries experience level, the majority (80.9%) exhibited a very low level (DMFT <5.0), while only a small proportion presented moderate (4.8%) or high (0.8%) levels of caries experience. In terms of oral hygiene status, the mean DI‐S score among primary caregivers was 0.97 (±0.62). Most caregivers (*n* = 170, 67.7%) were rated as fair, 64 (25.5%) had good oral hygiene, and only 6.8% had poor oral hygiene.

**Table 2:** Oral health status of the recruited primary caregivers (*n* = 251).

Oral health status	Mean (SD)	*n* (%)
Caries status		
DMFT	2.62 (3.31)	—
Caries prevalence (DMFT > 0)	—	180 (71.7)
Level of caries experience		
Very low (DMFT <5.0)	—	203 (80.9)
Low (DMFT 5.0–8.9)	—	34 (13.5)
Moderate (DMFT 9.0–13.9)	—	12 (4.8)
High (DMFT >13.9)	—	2 (0.8)
Oral hygiene status		
DI‐S scores	0.97 (0.62)	—
Oral hygiene levels		
Good	—	64 (25.5)
Fair	—	170 (67.7)
Poor	—	17 (6.8)

### 3.3. Oral Health‐Related Behaviors Among the Recruited Primary Caregivers

Among the recruited primary caregivers, 78.5% (*n* = 197) brushed twice daily or more, and the majority (80.9%, *n* = 203) spent less than 3 min per toothbrushing session. Additionally, 17.9% (*n* = 45) of caregivers reported snacking 3 or more times daily (Table 3).

**Table 3:** Oral health‐related behaviors of the recruited caregivers (*n* = 251).

Oral health‐related behaviors	*n* (%)
Toothbrushing frequency	
Once daily or less	54 (21.5)
Twice daily or more	197 (78.5)
Toothbrushing duration	
<3 min	203 (80.9)
≥3 min	48 (19.1)
Snacking frequency	
<3 times daily	206 (82.1)
≥3 times daily	45 (17.9)

### 3.4. Cariostat Scores and Associated Factors Among the Recruited Primary Caregivers

The mean Cariostat score among the primary caregivers was 1.57 (±0.43). The majority (96%) of primary caregivers exhibited moderate to high risk, and only 4.0% (*n* = 10) of the caregivers had a Cariostat score below 1.0 (Table 4). The proportion of caregivers with Cariostat scores ≥2.0 was significantly higher among those with a DMFT score ≥5 than among those with a DMFT score <5 (45.8% vs. 30.0%, *p* = 0.037). A significantly higher proportion of caregivers with Cariostat scores ≥2.0 reported toothbrushing durations of less than 3 min (36.5% vs. 18.8%, *p* = 0.019). Regarding snacking frequency, caregivers who snacked 3 or more times per day had higher Cariostat scores (48.9% vs. 29.6%, *p* = 0.013). Additionally, grandparents showed higher scores than other family roles (*p* < 0.001). Caregivers caring for children with high Cariostat scores also exhibited significantly high Cariostat scores (57.6% vs. 25.5%, *p* < 0.001, Table 5).

**Table 4:** Cariostat test results among the recruited caregivers (*n* = 251).

Cariostat test result	*n* (%)	Mean (SD)
Cariostat score	—	1.57 (0.43)
Levels		
Low (0–0.5)	10 (4.0)	—
Moderate (1–1.5)	158 (62.9)	—
High (≥2.0)	83 (33.1)	—

**Table 5:** Factors associated with caregivers’ Cariostat scores (*n* = 251).

Variables	Cariostat scores		*χ* ^2^/*t*	*p*‐Value
	≥2.0	0–1.5		
	*n* (%)	*n* (%)		
Gender				
Male	27 (35.5)	49 (64.5)	0.298	0.585
Female	56 (32.0)	119 (68.0)		
Highest educational level				
High school or below	35 (36.8)	60 (63.2)	0.984	0.321
Bachelor’s degree or above	48 (30.8)	108 (69.2)		
Caries status				
DMFT <5	61 (30.0)	142 (70.0)	4.370	0.037
DMFT ≥5	22 (45.8)	26 (54.2)		
Oral hygiene status				
Good	22 (34.4)	42 (65.6)	0.153	0.926
Fair	56 (32.9)	114 (67.1)		
Poor	5 (29.4)	12 (70.6)		
Toothbrushing frequency				
Once daily or less	23 (42.6)	31 (57.4)	2.820	0.093
Twice daily or more	60 (30.5)	137 (69.5)		
Toothbrushing duration				
<3 min	74 (36.5)	129 (63.5)	5.497	0.019
≥3 min	9 (18.8)	39 (81.2)		
Snacking frequency				
<3 times daily	61 (29.6)	145 (70.4)	6.201	0.013
≥3 times daily	22 (48.9)	23 (51.1)		
Role in the family				
Mother	49 (29.5)	117 (70.5)	18.616	<0.001
Father	22 (31.0)	49 (69.0)		
Grandparents	12 (85.7)	2 (14.3)		
Cariostat scores of children				
Low or moderate (0–1.5)	49 (25.5)	143 (74.5)	21.019	<0.001
High (≥2.0)	34 (57.6)	25 (42.4)		

Table 6 presents the results of univariate and multivariate logistic regression analyses examining factors associated with high Cariostat scores among caregivers. In the univariate model, parents with a DMFT index ≥5 (OR = 1.97, 95% CI: 1.03–3.75), snacking ≥3 times per day (OR = 2.27, 95% CI: 1.18–4.40), grandparents (vs. father, OR = 13.40, 95% CI: 3.29–90.7), and children under care with high Cariostat scores (OR = 3.97, 95% CI: 2.17–7.37) exhibited significantly higher odds of Cariostat scores ≥2.0. Brushing teeth for ≥3 min (OR = 0.40, 95% CI: 0.18–0.84) was associated with a significantly reduced risk, while brushing ≥ twice per day (OR = 0.59, 95% CI: 0.32–1.10) showed a nonsignificant protective trend, and maternal caregiving role (OR = 0.93, 95% CI: 0.51–1.72) was not associated with Cariostat results.

**Table 6:** Univariate and multivariate logistic regression models identify factors associated with high‐level Cariostat scores among caregivers.

Covariates	Univariate model		Multivariate model	
	Crude	*p*‐Value	Adjusted	*p*‐Value
	OR (95% CI)		OR (95% CI)	
Caries status (DMFT)				
≥5 (Ref: <5)	1.97 (1.03–3.75)	0.039	2.31 (1.08–4.94)	0.030
Toothbrushing frequency/day				
≥Twice (Ref: ≤ Once)	0.59 (0.32–1.10)	0.095	0.64 (0.31–1.33)	0.227
Toothbrushing duration				
≥3 min (Ref: <3 min)	0.40 (0.18–0.84)	0.022	0.47 (0.19–1.07)	0.084
Snacking frequency/day				
≥3 times (Ref: <3 times)	2.27 (1.18–4.40)	0.014	2.96 (1.41–6.28)	0.004
Role in the family				
Mother (Ref: Father)	0.93 (0.51–1.72)	0.821	0.75 (0.39–1.46)	0.399
Grandparents (Ref: Father)	13.40 (3.29–90.7)	0.001	9.65 (2.18–68.70)	0.007
Cariostat scores of children under care				
High (Ref: Low/moderate)	3.97 (2.17–7.37)	<0.001	4.05 (2.09–7.97)	<0.001

In the multivariate adjusted model, most associations remained consistent, namely, DMFT index ≥5 (OR = 2.31, 95% CI: 1.08–4.94), frequent snacking (OR = 2.96, 95% CI: 1.41–6.28), grandparental care (OR = 9.65, 95% CI: 2.18–68.70), and high Cariostat scores of children under care (OR = 4.05, 95% CI: 2.09–7.97) continued to predict elevated caries risk. The protective effect of longer toothbrushing duration was attenuated, whereas toothbrushing frequency (OR = 0.64, 95% CI: 0.31–1.33) and maternal caregiving role (OR = 0.75, 95% CI: 0.39–1.46) remained not significantly associated (Table 6).

## 4. Discussion

To the best of our knowledge, this cross‐sectional study was the first to investigate caries activity among caregivers of preschool children and to explore potential associated factors. One of our main findings was that caregivers of children with high Cariostat scores were more likely to have high Cariostat scores themselves (adjusted OR = 4.05, 95% CI: 2.09–7.97). This finding revealed a potential association in caries risk between children and their caregivers. Previous studies have consistently reported a positive association between caregivers’ and their children’s caries status, especially for mothers and children [16, 25]. One possible explanation is that caregivers’ own dental health predicts their oral health knowledge and behavior, which in turn influence the preventive oral health practices they apply for their children [26]. Additionally, cariogenic bacteria, such as *S. mutans*, can be transmitted from caregivers to children via shared tableware, kissing, blowing on food, and other forms of intimate contact [27]. Coupled with similar dietary habits within the family [28], a homogeneous cariogenic oral environment is formed, which collectively explains the strong association between caries risk of caregivers and their children. The findings support a family‐based strategy to caries prevention. Rather than focusing solely on the child, assessing cariogenic activity among caregivers may help identify shared risk factors and guide interventions targeting the family.

Caregivers’ caries status was significantly associated with their Cariostat scores. Caregivers with DMFT scores ≥5 were more likely to exhibit high Cariostat scores compared to those with DMFT scores <5 (adjusted OR = 2.31, 95% CI: 1.08–4.94). This finding aligned with previous studies reporting a positive association between caries experience and Cariostat scores [11, 23, 24]. The observed association suggests that individuals with more severe caries experience may continue to have a cariogenic oral environment, potentially characterized by a high level of cariogenic microbiota [29]. This may contribute to persistent cariogenic activity and, consequently, an increased risk of future caries development.

Furthermore, frequent consumption of between‐meal sugar‐containing snacks or beverages is associated with an increased risk for developing caries lesions [3]. In this study, caregivers who consumed snacks at least 3 times per day also tended to have Cariostat scores ≥2.0 (adjusted OR = 2.96, 95% CI 1.41–6.28, *p* = 0.004). In a cross‐sectional study, the dietary patterns of two groups of young adults were investigated, showing that the high caries group (with ≥5 decayed surfaces) had a significantly higher frequency of snack and total meal intake than the caries group with 2–4 decayed surfaces [30]. Therefore, Cariostat may serve as a valuable adjunct to conventional caries assessment in adult caregivers. Identifying caregivers with high Cariostat scores may help to implement targeted preventive interventions, such as dietary management, fluoride‐based preventive measures, and regular monitoring, rather than focusing solely on the treatment of existing carious lesions.

In our study, a high Cariostat score was observed more in grandparents than among parents. Interestingly, preschoolers with grandparents as the primary caregiver were associated with a higher risk of ECC compared with preschoolers with a mother or nursery school employees [31]. Parental oral health literacy can influence their children’s oral health, and it is defined as “the caregiver’s ability to access, understand, and use healthcare information to make appropriate decisions about their children’s oral health [32].” Moreover, parents’ toothbrushing practices, such as frequency and techniques, could influence their children’s oral hygiene [33]. Reinforcing the importance of early oral health education to caregivers and parents can reduce the risk of ECC in children.

Several limitations existed in this study. Firstly, the caregivers were recruited using a convenience sampling approach. We included mothers, fathers, and grandparents, but only 5.6% of children were cared for by grandparents. Although the observed risk trend for grandparents as caregivers was clinically relevant and consistent with the literature, the small number of grandparent caregivers in our sample (*n* = 14) resulted in wide CIs for the subgroup analysis, reducing the precision of estimates and the statistical power to detect meaningful differences compared with parent caregivers (mothers: 66.1%, fathers: 28.3%). Therefore, the magnitude of the observed effect (OR = 9.65) should not be interpreted as a precise estimate of risk, and future studies with larger randomly selected cohorts are warranted. Moreover, oral hygiene status was assessed using DI‐S scores in this study, a simple and valid approach for evaluating debris on tooth surfaces, and no significant association was found between caregivers’ oral hygiene status and their Cariostat scores. However, the sensitivity of the DI‐S index (75.2%) was reported to be lower than that of the O’Leary plaque index (96.8%) and the oral hygiene index for community use (CPI, 95.1%) [34]. Further studies are warranted to verify these findings using more sensitive indices.

Another limitation was that Cariostat was the only CAT kit used in this study. The occurrence of dental caries is influenced not only by the acidogenic potential of microorganisms but also by dietary habits, fluoride exposure, oral hygiene practices, and socioeconomic factors [23, 35, 36]. Cariostat evaluated only one biological dimension of caries risk assessment and did not capture the full complexity of caries risk. In addition, cross‐sectional studies could not confirm causal relationships. More well‐designed prospective studies that use comprehensive caries risk assessment are warranted to determine the level of caries activity and its risk factors in the target population.

## 5. Conclusion

The principal findings of this study revealed that caregivers’ Cariostat scores were significantly associated with their own caries status, snacking frequency, relationships with preschool children, and the Cariostat scores of preschoolers under their care. Among those factors, the strongest association was observed for children’s Cariostat levels. Specifically, caregivers caring for children with high Cariostat scores were more likely to have high Cariostat scores (adjusted OR = 4.05, 95% CI: 2.09–7.97). The above findings highlight the close association between caregivers’ and children’s oral health conditions and suggest that family‐based oral health promotion strategies may be beneficial in supporting caries prevention among caregivers and children.

## Author Contributions

**Colin Tsz Wo Lee:** investigation, data curation, writing – original draft. **Sifan Wang:** formal analysis, writing – original draft. **Xinyi Zeng:** investigation and project administration. **Cynthia Kar Yung Yiu:** supervision, writing – review and editing. **Hai Ming Wong:** supervision, writing – review and editing. **Ni Zhou:** conceptualization, methodology, investigation, funding acquisition, writing – review and editing.

## Funding

The study was supported by a Seed Fund from the University Research Committee of HKU and a basic research fund from the Department of Science and Technology of Yunnan Province (Grant 202201AU070140).

## Disclosure

All authors have read and approved the final version of the manuscript. Corresponding author (Ni Zhou) had full access to all of the data in this study and takes complete responsibility for the integrity of the data and the accuracy of the data analysis.

## Ethics Statement

This study was reviewed and approved by the local Ethics Committee of the Anning Maternity and Child Care Hospital (Ref No. ANFY2024KQ001) and the Institutional Review Board of the University of Hong Kong/Hospital Authority Hong Kong West Cluster (Ref No. UW24‐822). Informed consent forms were signed by the participants prior to the commencement of this study.

## Conflicts of Interest

The authors declare no conflicts of interest.

## Data Availability

The datasets analyzed during the current study are available from the corresponding author on reasonable request.
